# Time-Resolved Soft X-ray Diffraction Reveals Transient Structural Distortions of Ternary Liquid Crystals

**DOI:** 10.3390/ijms10114754

**Published:** 2009-11-04

**Authors:** Wilson Quevedo, Christian Peth, Gerhard Busse, Mirko Scholz, Klaus Mann, Simone Techert

**Affiliations:** 1 Max Planck Institute for Biophysical Chemistry, Am Fassberg 11, 37077 Göttingen, Germany; E-Mails: wqueved@gwdg.de (W.Q.); gbusse1@gwdg.de (G.B.); mscholz5@gwdg.de (M.S.); 2 Laser-Laboratorium Göttingen e. V., Hans-Adolf-Krebs-Weg 1, 37077 Göttingen, Germany; E-Mails: christian.peth@llg-ev.de (C.P.); kmann@llg-ev.de (K.M.)

**Keywords:** liquid crystal, soft X-rays, time resolved X-ray scattering

## Abstract

Home-based soft X-ray time-resolved scattering experiments with nanosecond time resolution (10 ns) and nanometer spatial resolution were carried out at a table top soft X-ray plasma source (2.2–5.2 nm). The investigated system was the lyotropic liquid crystal C_16_E_7_/paraffin/glycerol/formamide/IR 5. Usually, major changes in physical, chemical, and/or optical properties of the sample occur as a result of structural changes and shrinking morphology. Here, these effects occur as a consequence of the energy absorption in the sample upon optical laser excitation in the IR regime. The liquid crystal shows changes in the structural response within few hundred nanoseconds showing a time decay of 182 ns. A decrease of the Bragg peak diffracted intensity of 30% and a coherent macroscopic movement of the Bragg reflection are found as a response to the optical pump. The Bragg reflection movement is established to be isotropic and diffusion controlled (1 μs). Structural processes are analyzed in the Patterson analysis framework of the time-varying diffraction peaks revealing that the inter-lamellar distance increases by 2.7 Å resulting in an elongation of the coherently expanding lamella crystallite. The present studies emphasize the possibility of applying TR-SXRD techniques for studying the mechanical dynamics of nanosystems.

## 1. Introduction

**Figure 1.**
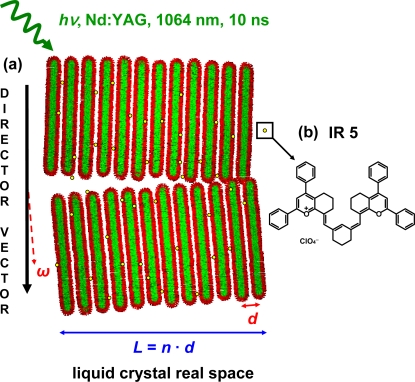
(a) Schematic presentation of the liquid crystal microscopic structure for two crystallites in real space. The lamellae are represented as rods with hydrophobic carbon chains (green) and polar heads (red). The periodic arrangement of the system is defined in terms of *d* = inter-lamellae spacing, *L =* crystallite thickness and ω = angle between director vector and normal vector. (b) Infrared sensitive laser dye IR 5.

Liquid crystal compounds have been under study for more than a century since their discovery by Reinitzer [1] in 1888. Due to the remarkable physical-chemical properties these materials are used in a wide range of applications [2,3]. In this study, a range of liquid crystal phases appears when the used non-ionic surfactant agent, an alcohol ethoxylate (AE), is dispersed in a ternary mixture of both polar and non-polar solvents and oil at different temperatures and surfactant concentrations. From a chemical point of view, the non-ionic surfactant agents have a rather pronounced amphiphilic character, consisting of two well defined “molecular regions”, a highly hydrophilic oligoether head and a long hydrophobic alkyl chain. These features enhance the wetting and foaming properties of the system by lowering the interfacial tension between the two media or interfaces. This allows the use of non-ionic surfactants for a wide range of applications in the food industry [4–6], as detergents [7], as fertilizers [8,9] and even as enhancers for the effectiveness of the diffusion of active ingredients within plants [10,11]. From a structural point of view, the formation of ordered structures of macroscopic size with nanoscale periodicity or lower is of great interest since it does play a key role in nanotechnology sciences. A good example is the process of molecular self-assembly, which has been under intensive research due to its use as a ‘tool’ for construction of different micro- and nanostructured materials [12–15]. In this direction, liquid crystal nanostructures are presented as a highly interesting class of chemically inspired nanostructures [16,17]. The system of investigation is a ternary mixture composed of C_16_E_7_/paraffin/glycerol/formamide and is photo-sensitized with the infrared laser dye IR 5 [18]. Our past studies show the possibility to photo-induce phase transitions in similar ternary lyotropic liquid crystal systems [19] upon optical excitation. In this work, the focus is on the stability of structural liquid crystal nano-ensembles formed within the ternary mixture. The main interest is the investigation of the mechanical and elastic properties of the liquid crystal phase, which are altered by the incoming optical pulse as a result of optical light absorption by the laser dye IR 5 and the system itself. Modifications of both properties occur through energy dissipation within the nano-ensemble inducing strain processes which result in fluctuations and undulations of the periodicity of the liquid crystalline phase (Figure 1).

**Figure 2.**
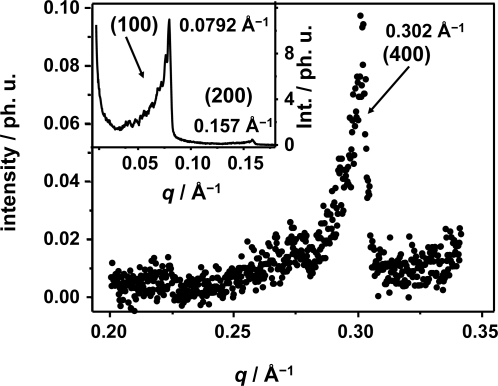
Experimental small angle X-ray scattering (SAXS) pattern for the ternary mixture C_16_E_7_/paraffin/glycerol/formamide at room temperature. The main graph shows the (400), as measured with the apparatus extension. The inset graph shows the (100) and (200) Bragg reflection peaks.

The periodicity of the liquid crystal is defined in terms of the distance between lamellae *d*, the thickness *L* of the crystallite which is given by the number *N* of stacking lamellae and the deviation, or azimuthal angle ω, of the nano-ensembles with respect to the director vector or main direction of orientation in the L_α_ phase. The periodic structure of the lamella “stacks” forming the nano-ensemble can be monitored by X-ray diffraction methods as the periodic electron density around the polar head of the surfactant agent gives rise to Bragg diffraction peaks with the Miller indices (100), (200) and (400). The small angle X-ray scattering pattern (SAXS) of the lamellar phase of the ternary mixture C_16_E_7_/paraffin/glycerol/formamide is shown in Figure 2. The main graph shows the profile of the (400) Bragg diffraction peak and the inset graph shows the diffraction peak assigned for the Miller indices (100) and (200). The (100), (200) and (400) Bragg reflections correspond to a *q* value of 0.0792 ± 0.008 Å^−1^, 0.1576 ± 0.004 Å^−1^ and *q* = 0.3021 ± 0.002 Å, respectively. The error reflects the varying Bragg diffraction intensity profile leading to different halfwidths in reciprocal space, with a broader distribution for the first order Bragg diffraction peak. A *q* value of *q* = 0.0792 Å^−1^ of the Miller indices (100) corresponds to a *d*-spacing of 79.4 Å as indicated on Figure 1.

The time-resolved diffraction studies on lyotropic liquid crystal phases rely on the long range order effects (*d* = 79.4 Å) arising from the described periodicity of the system. The structure of this lyotropic liquid crystal phase has been identified with a smectic A L_α_ phase. The natural classification of this phase is denoted as $G_{1}^{3}$. It describes the dimensionality of the liquid crystal object with periodicity in one direction. As the periodicity occurs on the nanometer scale it gives rise to a Bragg peak in the small angle regime. In the XUV regime the broadened elongated Bragg reflection arises as a consequence of the nanoscale periodicity of the system in one spatial coordinate.

Complementary, the spectra of the lyotropic liquid crystalline phase do resemble the position and shape of the peaks to that of the pure surfactant SAXS pattern, strongly suggesting a highly ordered lamella formation under the conditions of preparation.

The aim of the present study is to obtain a detailed picture of the dynamical behavior of the crystallite lamella nano-ensembles. Time-resolved X-ray diffraction (TR-XRD) allows a detailed study of the time evolution of structural intermediates and short living states of chemical systems at wide range of time-scales [20,21]. In a TR-XRD experiment structural changes of a sample can be investigated by the well established pump-probe scheme, in which a laser (pump) is synchronized to an X-ray source (probe) [22,23]. By varying the time delay between optical laser pump pulse and X-ray probe pulse information about the time evolution of structural changes in the photo-excited matter is obtained and can be analyzed by X-ray diffraction techniques. Optical laser excitation guarantees a coherent excitation of the sample and the possibility of a controlled energy deposition in the system in a controlled interval of time. Thus, it is possible to trigger optical induced structural changes in the sample at a well defined time point zero. In this sense, experiments with high temporal as well as spatial resolution and short data acquisition times with sufficient signal-to-noise ratio require X-ray sources of high brilliance such as third generation synchrotron sources [24]. Parallely, the ongoing progress in the development of laboratory-scale X-ray sources enables experimental techniques that were performed almost exclusively at synchrotron sources. Table-top soft X-ray sources of high brilliance, such as laser-produced plasmas [25,26], high harmonic radiation [27] or X-ray lasers [28] are now used for various applications, e.g., X-ray microscopy [29], lensless diffractive imaging [30], photoelectron spectroscopy [31] or absorption spectroscopy [32]. Investigation with laser-plasma soft X-ray sources offers the unique possibility to study samples with periodic structures in the nanometer range, as their periodicity matches the Bragg condition for the experimental wavelengths.

**Figure 3.**
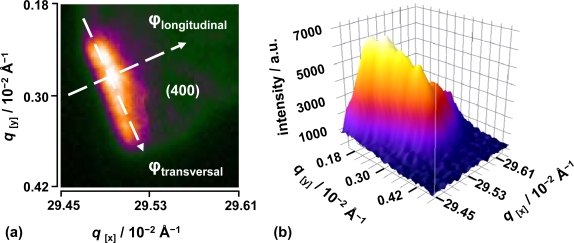
(a) Two-dimensional signal of the (400) Bragg peak recorded in reflection mode. The CCD images were background corrected and the integrated intensity was normalized to the incident flux monitored with the XUV normalization unit. (b) Three-dimensional plot of the diffraction signal. The scales are given in reciprocal *q*-space. The Bragg peaks are transversally elongated due to the finite individual lamellar crystallite size and due to the broadband properties of the soft-x ray source (Laue beam).

**Figure 4.**
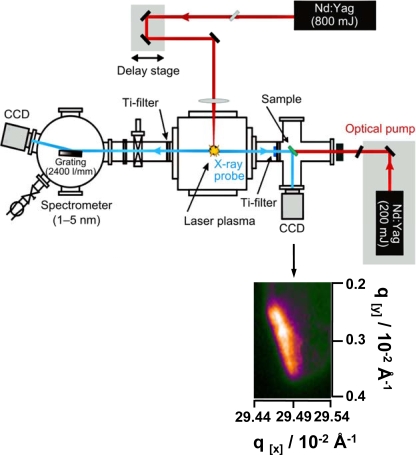
Experimental setup for the time-resolved soft X-ray diffraction experiments. The soft X-ray probe pulse is delivered by a laser-driven plasma source. For the time delay a second Nd:YAG laser is electronically synchronized to the X-ray pulse. The diffraction signal-to-noise ratio is enhanced by using a 2 × 2 pixel binning.

For a complete description of the presented setup refer to the experimental section (Figure 4). The presented diffraction pattern is assigned to a crystallite nano-ensemble of stacked lamellae. The probe pulse is polychromatic, the sample polycrystalline and its recorded Bragg diffraction spot appears rather broadened and elongated as the experiment has been performed in reflection mode. The experimentally obtained diffraction signal is shown in Figure 3.

The diffraction analysis of the polycrystalline ensemble was carried out as function of the diffraction angle 2θ defined here in the direction of the ϕ_longitudinal_ line and the degree of orientation of the lamellar planes of the crystallite ensemble, or the azimuthal angle (ω) extension ϕ_transversal_, with respect to the director vector [33]. By analysis of the integrated intensity profile, along the defined ϕ lines, the Bragg diffraction orientation and position changes as function of time can be well analyzed. On one hand, ϕ_longitudinal_ provides information on the structural lattice periodicity of the L_α_ phase. The lamella-lamella distance *d* = 2π/q is monitored as a function of time providing a “molecular movie” of the dynamics of the system as is intrinsically related to the structure factor. On the other hand, φ_transversal_ is related to the azimuthal angle ω as it provides information on the deviation angle of the periodic lattice with respect to the *director vector*. Movement along ω is as well monitored as a function of time, providing structural information normally related to distortion and shearing effects within the *N* lamella planes contained within a crystallite. The experimental conditions of the source produce similar results as if a rocking curve was obtained since the broadband properties of this Laue-like XUV radiation allows for resembling the diffraction over the entire wavelength range (24–50 Å). It should be remarked that the investigated (400) Bragg diffraction peaks are transversally elongated due to the finite individual lamellar crystallite size and due to the Laue properties of the soft X-ray radiation source.

## 2. Experimental Section

The experimental setup for time resolved soft X-ray diffraction experiments with a laser-driven plasma source is shown schematically in Figure 4.

The experiments were carried out in a pump-probe experiment with stroboscopic illumination. For the generation of the soft X-rays in the spectral range of the “water window” (2.4–5.0 nm) that were used as probe pulse, a Nd:YAG laser beam (Innolas, 1064 nm, 1 Hz, 800 mJ, 7 ns) was focused into a pulsed gas puff target centered in a vacuum chamber. The laser focus has a diameter of about 60 μm, yielding power densities of up to 4 × 10^12^ W/cm^2^ that are sufficient to ignite a hot and dense plasma. Krypton is employed as target gas (backing pressure 25 bar), accomplishing broadband radiation (Kr XXV – Kr XXXVI) with pronounced maxima in the spectral range of the “water window”. Due to the small mean free path of the soft X-ray radiation at atmospheric pressure the target vacuum chamber is evacuated to approximately 10^−4^ mbar.

The plasma can be monitored with an XUV spectrometer (1–5 nm) consisting of a 100 μm entrance slit, an aberration corrected flat-field grating (Hitachi, 2,400 lines/mm) and a back-side illuminated CCD camera (Roper Scientific, 2048 × 512 pixel, 13 μm pixel size). The spectrometer was mounted 90° to the laser beam and opposite to the diffraction experiment. To block visible radiation from the plasma and scattered laser light a titanium foil (200 nm thickness) was positioned in both branches of the setup. The distance between plasma source and sample was 160 mm and the distance between sample and detector was 135 mm.

The diffraction pattern was recorded in reflection geometry with a back-side illuminated CCD camera (Roper Scientific, 1,024 × 1,024 pixel, 13 μm pixel size). To enhance the signal-to-noise ratio a 2 × 2 pixel binning was used.

For adjustment the samples were mounted on a rotary/linear motion stage. An XUV pulse length of about 4 ns was measured with a high-speed X-ray photodiode (AXUV HS 5, rise time 700 ps).

To investigate different time scales of the sample perturbation, the optical pump pulse was generated by coupling a second Nd:YAG laser (Innolas, 200 mJ, 7 ns) with time delays between 20 ns and 1 s. The laser was synchronized electronically to the other laser system with the help of a pulse/delay generator (Stanford Research Systems). In this configuration a temporal jitter of about ±10 ns was realized.

The pump energy on the sample was adjusted by using several neutral density filters so the pulse energy of the optical pump on the sample was between 5–7 μJ. Each time point was obtained on a stroboscopic mode over an average of 100 shots. Furthermore, CCD images were background corrected and the integrated intensity was normalized to the incident flux monitored with the XUV spectrometer.

### 2.1. Preparation of the Lyotropic Liquid Crystals

The investigated systems can be described as function of two variables: temperature and surfactant concentration. The surfactant volume fraction defined as γ = *m*_surfactant_/(*m*_polar solvent_ + *m*_oil_ + *m*_surfactant_) was maintained at 42%. At this surfactant concentration the system is in the solid state until 45 °C. The ternary solutions were prepared from a mixture of glycerol and formamide (90:10 respectively, Merck, polar solvent), paraffin (Fluka, non polar solvent), and the non ionic surfactant agent, heptaethylene glycol monohexadecyl ether, CH_3_(CH_2_)_15_(OCH_2_CH_2_)_7_OH (C_16_E_7_, Fluka, 98% purity), used without further purification. The mixture was stirred at room temperature for 1 hour in a thermostat bath and then the liquid crystal phase was identified using the polarized screening technique (PLS).

Then the samples were placed on Mylar thin-films by using a spin coating method (4,000 rev/min, P-600 Spin Coating Systems) with an estimated sample thickness of 50 μm. The calculated penetration depth of the soft X-rays for the lyotropic liquid crystals phase is between 0.7–1 micrometers, which is much smaller than the sample thickness (50 μm). Therefore, only 0.7–1 μm of the whole sample has been probed with the XUV radiation. For the laser system and dye-sensitizer used, the temperature jump was estimated to be between 15–21 K.

All the samples were vacuum tested (10^−5^ mbar) with significant stability for all the systems. The sample holder had a ring like shape, so that it was possible to illuminate the samples from the backside with the optical pump pulse.

## 3. Results and Discussion

**Figure 5.**
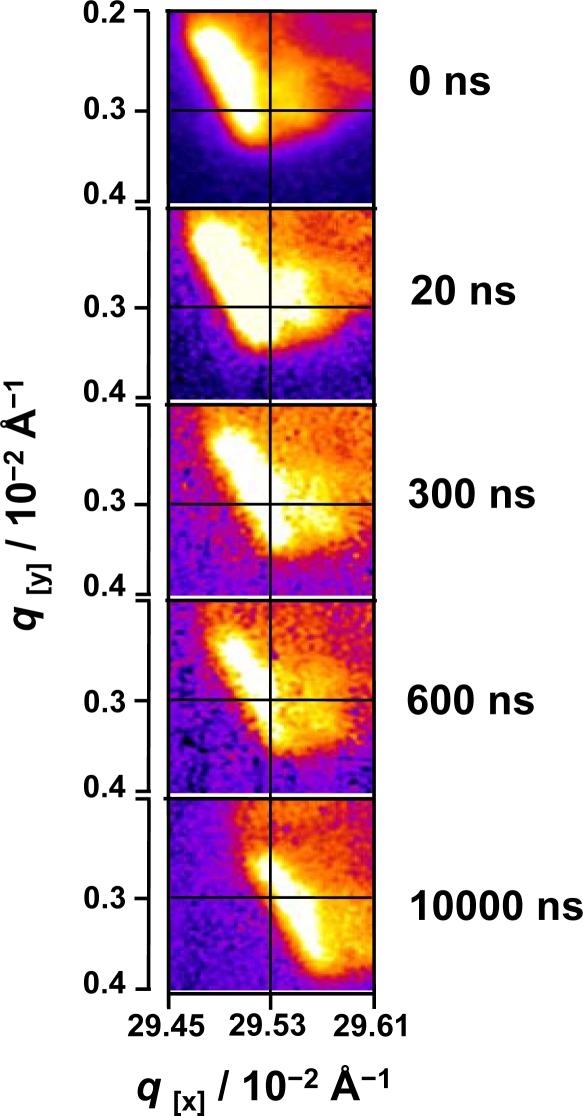
Time-resolved Bragg diffraction spot. The diffraction signal is displaced coherently in both *q*-axes, within the first 600 ns.

It is well known that the interaction of external fields with the described systems causes susceptibility changes in these soft ordered materials like induced alignment and ordering effects [34]. The ternary mixture is dye-sensitized, so the optical infrared pulse excites solid matter coherently introducing a heat-jump on the system. The excitation mechanism has already been extensively studied on similar lyotropic liquid crystals [19]. Note that in the present case, the excitation conditions are well within the Stokes regime leading to a highly efficient energy transfer from the dye to the surrounding liquid crystal following a temperature jump scheme. The energy deposition of these processes is regarded as a very fast heat deposition and transfer within the system. By inspection of the diffraction pattern of the liquid crystal system at different time scales (Figure 5) a pronounced change of the absolute position of the Bragg diffraction is observed within the first 10 μs. The total displacement of the reflection with respect to its maxima along the *q*-axis is estimated to be around 0.005 Å^−1^ in reciprocal space. Furthermore, the movement of the reflection occurs in a coherent way so the full reflection is displaced at the same time and equal number of pixels along both *q*_x_ and *q*_y_ axes, which are the given scales in Figure 5. The absolute movement starts within the first few hundred nanoseconds and it fully develops in the microsecond timescale, as expected. The intensity of the diffraction peak is corrected against the total intensity of the plasma source pulse which was monitored online. Each time point is obtained in a stroboscopic mode over an average of 100 shots.

**Figure 6.**
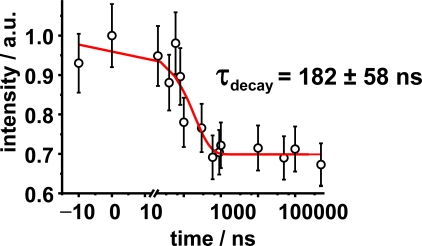
Time-resolved integrated intensity of the Bragg spot. The decay follows a mono-exponential decay with a time constant of 196 ns. The decrease of intensity was analyzed by the Patterson analysis.

In addition, in Figure 6 the diffraction intensity of the Bragg spot was plotted as a function of time. The Bragg diffraction analysis is interpreted in terms of changes of the Fourier transformed diffraction intensity from reciprocal space into real space, before and after optical excitation. The recorded negative time point fluctuates in average by 8%. The total intensity decreases about 30% suggesting a strong change in periodicity within the nano-ensemble lattice. The decay follows a mono-exponential decay law with a time constant of 182 ns. Before optical excitation, the thermally stabilized structure has a periodicity of 79.4 Å between polar hydrophilic heads. As the optical pump excites the sample, the absorbed photons create a disordered intermediate where the distance within two scattering centers is increased by almost ~2.7 Å, as revealed by Patterson analysis. The decrease of the diffracted intensity for the photo-created structure does not recover at longer time scales (μs).

So with a time window limited to ~182 ns, the intensity decrease is compared to acoustical phonons propagating along the L_α_ phase. Theory states [35] that sound propagation in smectic liquid crystals will occur with 2 sound waves with speeds designated as ν_1_, ν_3_ and 3 elastic constants: *C*_11_*, C*_33_ and their combination *C*_13_. These constants can be physically understood in terms of the different manners in which the lamellae ensemble can dissipate the sound wave throughout its structure. For simplicity, it is assumed in the following analysis that upon optical excitation the propagation of the sound wave does occur in a coherent manner. This assumption is supported by the fact that the Bragg peak does move coherently and similar distances along both *q*_x_ and *q*_y_ axes. Moreover, previous results suggest for similar lyotropic liquid crystal phases [36] that their elastic constants become *C*_11_ = *C*_33_ = *C*_13_ and *C*_b_ = ν_1_ = ν_3_. Here, the expression for the propagation speed *v* of the acoustic wave is rewritten as:

(1) $$C_{b}\;=\;C_{\text{long}}\;+\;C_{\text{trans}}$$

where *C_b_* is expressed as the sum of the longitudinal mode $C_{\text{long}}\;=\;\sqrt{E/\rho_{LC}}$, *E* as Young’s modulus and the transversal mode as $C_{\text{trans}}\;=\;\sqrt{G/\rho_{LC}}$, with *G* as the shear modulus, and *ρ_LC_* as the ternary mixture density for both moduli. Since the propagation is considered to be isotropic, both Young’s and shear moduli do have the same value, obtained from literature [36,37]. The transversal mode *C_trans_* describes the part of the acoustic wave propagating perpendicular to the lamella surface and is identified with the elastic constant *C*_11_. The longitudinal mode, *C_long_* describes the part of the acoustical wave displacing along the (400) plane corresponding to the *C*_33_ elastic constant. The sound wave *C_b_* propagates with a speed of 1791 m/s in a coherent manner through both *C* directions. As the time decay for the diffraction intensity is τ = 182 ns the acoustic waves will propagate 350 μm across the sample surface. Taking the XUV penetration depth into account (1 μm), only the movement across the surface could be fully monitored. In other words, acoustical phonons in both *C*_11_ and *C*_33_ directions propagate across the sample surface for several micrometers, thus inducing pronounced structural changes in the L_α_ phase periodic lattice. As the repetition rate of the apparatus is 1 Hz, the negative time point at – 10 ns corresponds to the intensity of the reflection after 0.99999999 seconds after irradiation with the Nd:YAG laser pulse. It emphasizes the recovery of the intensity at long time scales (sec).

Complementary, as the obtained diffraction signal accounts for the periodic lattice within the L_α_ phase, the structural effect caused by the propagation of the acoustic wave in this direction can be well described applying the Patterson analysis. The analysis of the relative displacement within the periodic lattice of the L_α_ lamellae was carried out on the corresponding (400) Bragg diffraction peak. According to this analysis the electron distribution *ρ(u)* in one dimension, here the one of the periodic arrays of lamellae is defined as:

(2) $$\rho (u)\;=\;(1/a)\left[F_{0}+2\sum_{h}F_{h}\text{cos}(2\pi hu/d)\right]$$

with *d* = lattice constant between the lamellae, *u* = relative coordinate of the electron density centres, *F*_0_ = predicted structure factor and *F_h_* = experimental structure factor, where *F_h_^2^* is directly proportional to the scattered integral intensity. The photo-induced structural changes are analyzed in a way in which the scattering centers are placed in the junction point of the polar heads of the surfactant agent as seen in Figure 1. The relative displacements for the decrease of intensity shown in Figure 6 are tabulated in Table 1.

**Figure 7.**
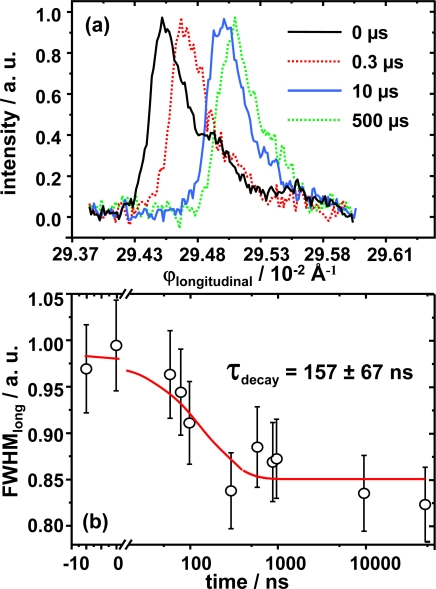
(a) Normalized integrated intensity FWHM, for several time scales (b) FWHM for ϕ_longitudinal_ as a function of time. The decay is well fitted with a mono-exponential decay with a lifetime of 157 ns.

To further refine the dynamical picture, the results of the relative electron distribution analysis are complemented with a detailed analysis of the halfwidth and displacement for both ϕdirections. The movement of the Bragg diffraction along the longitudinal direction is shown in Figure 7. This displacement is quantified on reciprocal space along the 2θ direction and estimated to be around Δ*q* = 0.005 Å^−1^ which corresponds to an increment of the diffraction angle Δ2θ = 0.16°. The movement along ϕ_longitudinal_ is an absolute movement and must not be confused with the relative displacement calculated by means of the Patterson analysis. The normalized value for the FWHM is shown as a function of time Figure 7 (b). The change of almost 20% in the FWHM value with respect time zero, strongly suggests some kind of photo-induced process within the lamella as it value decreases. By analyzing the curves FWHM versus time of Figure 7 (a) it is possible to extract structural information concerning the thickness and number of lamellae within the crystallite. The curve corresponds to the intensity profile along ϕ_longitudinal_ and its FWHM is analyzed in terms of the Scherrer equation. From this analysis one can extract information of the crystal size as the position of the maximum depends on the inter-lamellar distance, and the FWHM depends on the ordering and number of lamellae contained within the nano-ensemble. We consider the reflection to belong to a crystallite in the form of a thin plate-like ensemble of large area consisting of *N* lattice planes (lamellae) of spacing *d*. The Scherrer equation is given by:

(3) $$\Delta 2\theta \;=\;\frac{0.9\;\cdot \;\lambda }{L\;\cdot \;\text{cos}\theta_{0}}$$

where λ = 28.3 Å is the incoming wavelength matching the Bragg condition, *L* is the thickness of the crystallite nano-ensemble and cos θ_0_ is the cosine of half the Bragg angle of the experimental diffraction signal. From this formula the *L* value is obtained and equal to *L = N d* (Figure 1). Results for Scherrer analysis are presented in Table 1 as well.

**Figure 8.**
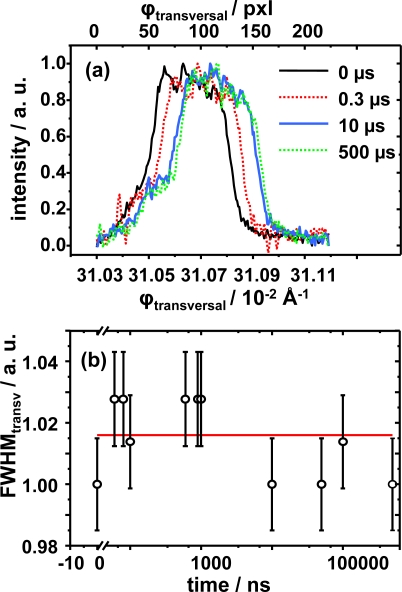
(a) Normalized intensity profile along the ϕ_transversal_ as a function of pixel displacement. (b) FWHM values along the ϕ_transversal_ as a function of time.

The next logical step is to monitor the movement and intensity changes along ϕ_transversal_ to fully refine the dynamics of the system. As stated before, the analysis of the FWHM in this direction provides information on the shearing process within the lamellae contained in the crystallite nano-ensemble, with the position changes indicating the angle of deviation with respect to the director vector. The FWHM as a function of time and the intensity profile position changes along ω are presented in Figure 8. It clearly shows that the movement along this direction is proportional and almost equal to the one on the 2θ direction. Note by inspection of Figure 8 (b) that the FWHM values of the intensity profile as a function of time, the main difference with respect to ϕ_longitudinal_, do not change with time. So the absence of a pronounced ordering-disordering process within the lamellae in the ω direction is suggested as the FWHM values do not vary more than 3% above the mean, which might indicate that disorganization-organization processes only occur along ϕ_longitudinal_. It also should be remarked again that the shape of the FWHM intensity profile is not a perfect Gaussian shape due to the transversally finite size of the individual lamellar crystallite sample and Laue broadband source used.

**Figure 9.**
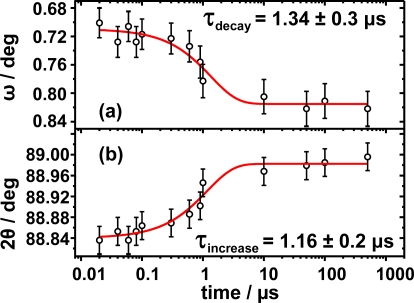
(a) Movement along ϕ_transversal_ as function of time. (b) Movement along ϕ_longitudinal_ as function of time. Both characteristic time scales indicate that the movement in both directions is a diffusion controlled process.

The absolute movement along both ϕ_longitudinal_ and ϕ_transversal_ as a function of time is presented in Figure 9. On one hand, the movement along 2θ was well fitted with a mono-exponential function and a characteristic rise time of τ_increase_ = 1.16 μs. On the other hand, the movement along ω was also fitted with a mono-exponential law and a characteristic rise time of τ_decay_ = 1.34 μs. The total movement along ϕ_longitudinal_ is Δ2θ = 0.16°, whereas the absolute value for increment along ω is slightly smaller with Δω = 0.12°. Remarkably, the movement along both directions occurs at similar time scales, within 1 microsecond, confirming the fact that both movements are diffusion controlled processes. It is also in good agreement with the assumption that the propagation of the acoustical phonons is rather coherent as the obtained microscopically displacement occurs at similar lengths and times along both directions.

The calculations for both displacement functions were done following a standard procedure [38]. The designation of the axes {**x**, **y**, **z**} is defined as follows, **z** is the direction of the direct beam, **x** is collinear with the detector normal and **y** is collinear with the detector’s slow coordinate (Y) (both at 2θ = 90°). The origin is located where the direct beam strikes the sample. The negative symbol for the displacement along the transversal mode is due to the arbitrary election of the axis direction of the displacement along the CCD.

**Figure 10.**
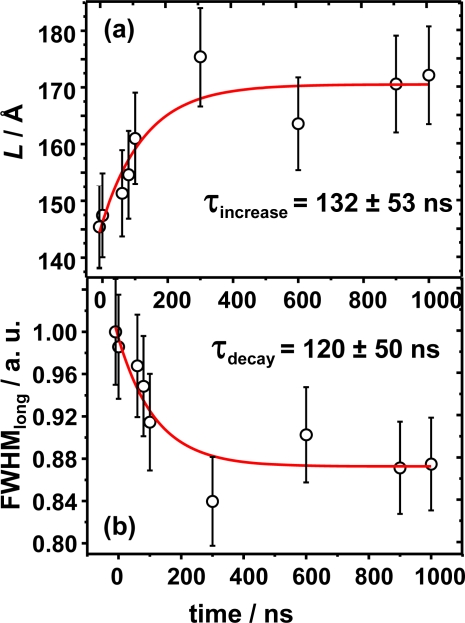
(a) Time-resolved evolution of the crystallite thickness. The thickness of the crystallite does increase for 20% of its original value. (b) Time evolution of the FWHM along the longitudinal direction.

As the FWHM varies on the ϕ_longitudinal_, the Scherrer analysis will be focused on the 2θ direction. The thicknesses of the crystal and the longitudinal FMHW as a function of time are summarized in Figure 10. The time evolution of the integrated FWHM along ϕ_longitudinal_ direction elucidates a fast process. This has been identified with a non-thermal photo-induced increase of the *d-*spacing within the crystallite nano-ensemble as the value of the FWHM decreases by almost 20%. Consequently a maximum thickness of *L* = 185 Å is yielded which represents an increase of 35 Å when compared to the thermally equilibrated structure. Note, that the number *N* of lamellae remains constant (Table 1). The time constant for this process, T = 120 ns, was obtained after fitting with a mono-exponential law. The energy dissipation which is monitored by the FWHM changes is also reflected in an increase of the thickness *L* to a more disordered phase. This might support the idea that energy dissipation occurs through its acoustical mode *C*_33_.

## 4. Conclusions

**Figure 12.**
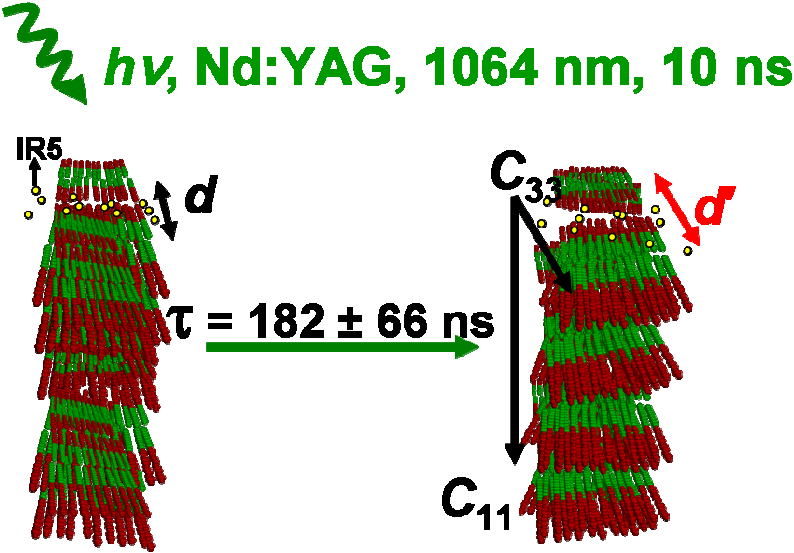
Schematic representation of the proposed mechanism. Upon optical excitation periodic organization occurs. However, the increased *d*-spacing diminishes the integral diffracted intensity. **C_11_** and **C_33_** are the acoustical modes along the transversal and longitudinal directions and the yellow dots the laser dye IR5.

Time resolved soft X-ray diffraction studies on lyotropic liquid crystals were performed at a home built plasma source. The ternary lyotropic liquid crystal system under study shows two well defined structural responses at different time scales: a structural photo-induced process in the ϕ_longitudinal_ direction and an absolute coherent macroscopic movement in both ω and 2θ directions. The mechanism suggested for the structural response is described in Figure 12. At the start, the sample order is not well defined with several lamellae stacked in a random manner within a mono-crystallite domain. As the system absorbs the energy upon optical excitation, it is proposed that the energy transfer occurs along the acoustic modes in an anisotropic manner as theory suggests [15]. Therefore the optical pulse decreases the total intensity of diffraction so that the number of scattering domains is either reduced (melting) or out of phase. The decrease in diffracted intensity occurs within 182 ns. As the used laser power is rather low (5–10 μJ) and the temperature jump calculated corresponds to a maximum of 15–21 K, the possibility of melting the sample is fairly low. Instead, the creation of a meta-stable phase is suggested, as it is known that the smectic phase is stable within a broader temperature range than the temperature jump caused by the laser and that the intensity does not recover at latter time scales (μs). However, the total diffracted intensity does recover at much longer time scales (sec).

Since the IR centers are placed within the polar heads it seems feasible that upon optical excitation, the energy dissipation process of the IR dye will affect the periodicity of the lattice. The decrease in intensity is explained in Figure 12. First the inter-lamellae *d*-spacing increases for almost ~2.7 Å as revealed by Patterson analysis. As the *d*-spacing increases it falls out of phase decreasing the total diffracted intensity. Note that the number of stacked *N* lamellae does not increase significantly. So, the creation of a fast photo-created intermediate with a decay time of 182 nanoseconds is proposed. The decay of intensity seems to be in relatively agreement with the decay time of the FWHM values along the ϕ_longitudinal_ intensity profile, which take place at about 120 nanoseconds. There was no indication of any fast photo-induced process along the ϕ_transversal_ direction as the halfwidth values of the intensity profile as function of time remains unchanged.

Also, our experiments show a macroscopic movement of the Bragg reflected peak along both transversal and longitudinal directions in a coherent manner and with similar time scales and vaguely different angular displacement. The macroscopic movement reveals an absolute displacement in 2θ of 0.16° and in ω of 0.12° with respect to the director vector. The movement along both directions is diffusion controlled and occurs within the first microsecond. As there is no evidence of energy dissipation along the ϕ_transversal_ direction and the absolute displacement is slightly different this might suggest the idea of small anisotropicity of the system. Nevertheless, more studies are planned to fully explain the structural response of the system at longer time scales and their anisotropic/isotropic behavior.

The possibility of carrying out home based studies in the XUV regime opens the door for time-resolved studies on biological systems which present periodicities in the nanometer scale. Those experiments are proposed for the future.

## Figures and Tables

**Table 1.** Results for Patterson and Scherrer analysis. In the first column the relative decrease of intensity at different times after optical excitation is depicted. The second column shows the change on the lattice periodic distance *u*. The third column presents the thickness of the crystallite at different times, and the 4^th^ column shows the number of lamellar planes which form the crystallite.

***Time delay* [ns]**	**Δ*I* / *I* [a. u.]**	**u [Å]**	***L* [Å]**	***N***
0	0	0	149 ± 0.49	1.9 ± 0.04
60	98 ± 8	0.66 ± 0.06	151 ± 0.50	1.9 ± 0.04
80	90 ± 7	1.52 ± 0.15	154 ± 0.51	1.9 ± 0.04
100	78 ± 6	2.23 ± 0.22	161 ± 0.53	2.0 ± 0.05
300	76 ± 6	2.31 ± 0.23	175 ± 0.58	2.1 ± 0.06
1000	72 ± 5	2.53 ± 0.24	172 ± 0.56	2.1 ± 0.05
10000	71 ± 5	2.54 ± 0.24	180 ± 0.58	2.2 ± 0.07
50000	68 ± 5	2.68 ± 0.25	184 ± 0.59	2.2 ± 0.07
100000	71 ± 5	2.54 ± 0.24	177 ± 0.58	2.1 ± 0.07
500000	68 ± 5	2.68 ± 0.25	170 ± 0.55	2.1 ± 0.06
*Average*		1.96 ± 0.19	167 ± 0.5	2.0 ± 0.05
